# Slow dynamics measured by phosphorescence lifetime reveals global conformational changes in human adult hemoglobin induced by allosteric effectors

**DOI:** 10.1371/journal.pone.0278417

**Published:** 2022-12-01

**Authors:** Gusztáv Schay, Judit Fidy, Levente Herenyi

**Affiliations:** Department of Biophysics and Radiation Biology, Semmelweis University, Budapest, Hungary; University of Alcalá, SPAIN

## Abstract

The mechanism underlying allostery in hemoglobin (Hb) is still not completely understood. Various models describing the action of allosteric effectors on Hb function have been published in the literature. It has also been reported that some allosteric effectors—such as chloride ions, inositol hexaphosphate, 2,3-diphospho-glycerate and bezafibrate—considerably lower the oxygen affinity of Hb. In this context, an important question is the extent to which these changes influence the conformational dynamics of the protein. Earlier, we elaborated a challenging method based on phosphorescence quenching, which makes characterizing protein-internal dynamics possible in the ms time range. The experimental technique involves phosphorescence lifetime measurements in thermal equilibrium at varied temperatures from 10 K up to 273 K, based on the signal of Zn-protoporphyrin substituted for the heme in the β-subunits of Hb. The thermal activation of protein dynamics was observed by the enhancement of phosphorescence quenching attributed to O_2_ diffusion. It was shown that the thermal activation of protein matrix dynamics was clearly distinguishable from the dynamic activation of the aqueous solvent, and was therefore highly specific for the protein. In the present work, the same method was used to study the changes in the parameters of the dynamic activation of human HbA induced by binding allosteric effectors. We interpreted the phenomenon as phase transition between two states. The fitting of this model to lifetime data yielded the change of energy and entropy in the activation process and the quenching rate in the dynamically activated state. The fitted parameters were particularly sensitive to the presence of allosteric effectors and could be interpreted in line with results from earlier experimental studies. The results suggest that allosteric effectors are tightly coupled to the dynamics of the whole protein, and thus underline the importance of global dynamics in the regulation of Hb function.

## Introduction

“Allostery” is a keyword in the description of the functioning of hemoglobin (Hb), yet its real mechanism is unknown to this day. The unanswered question is how a peripheral molecule and the hemes can interact from a rather long distance to change the oxygen binding affinity of the Hb. This question has been studied for a long time and remained in the center of interest. Several models have been developed to explain the functioning of Hb and the homotropic and heterotropic allosteric effectors. In our previous paper on HbA [1], we provided a concise overview of these models. Here is a reminder in the form of a short list of the most prominent ones:

“two-state allosteric model” or MWC model [2–4]
“stereochemical model” [5]
“sequential model” or KNF model [6]
“ensemble allostery model” or EAM [7]
“chemical perspective on allostery” [8]
“tertiary two state model” or TTS model [9]
“global allostery model” [10, 11]
“unified view about allostery” [12, 13].

**Fig 1:**
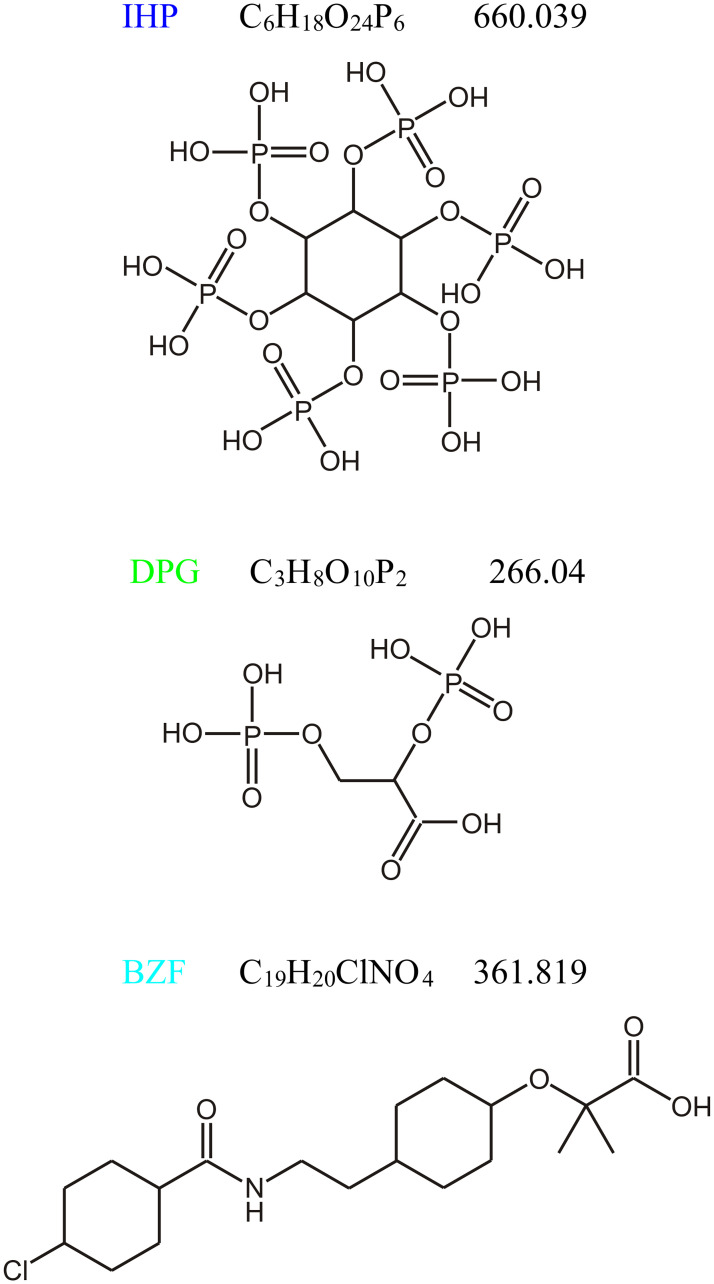
Chemical structure of the applied allosteric effectors (molar weights are also given): Inositol hexaphosphate (IHP), 2,3-diphosphoglycerate (DPG), bezafibrate (BZF).

Earlier it was also demonstrated by our research group that conformational changes induced by the binding of the heterotropic allosteric effectors Chloride ions (Cl^-^), Inositol Hexaphosphate (IHP), and Bezafibrate (BZF)–which can considerably reduce the oxygen affinity of Hb–affected the interdimeric interfaces of Hb in both conformational states, supporting the global allostery model [14, 15]. The chemical structure and the molar weight of IHP and BZF together with 2,3-diphosphoglycerate (DPG) are shown in Fig 1. The binding of these effectors is very different, and there is still controversy in the literature as to how and where they exactly bind. Cl^-^ and BZF are particularly not well understood, while it is known that IHP and DPG bind primarily through electrostatic interactions into the central cavity [16–18]. Based on the ensemble nature of allostery, it has been emphasized that there may not be a single set of conformational changes underlying allostery; rather, ligand binding may shift the protein’s conformational and dynamic ensemble, resulting in changes in thermodynamics and affinity at distant sites [7]. In this approach, continuum dynamics can span a rather broad timescale from ps up to ms. While the fast motions can be studied by experimental and computational methods, there is no proper adaptation of these techniques for longer time windows.

It has been discovered that the collisional quenching of tryptophan (TRP) fluorescence by diffusing molecular oxygen could be a marker sensitive to the internal fluctuations of the protein structure [19, 20]. This signal can report dynamic phenomena within the time frame of the TRP fluorescence lifetime, that is, in the ns time range. While this time frame allows for reaction with local atomic vibrations, the time window being in the milliseconds range of TRP phosphorescence would allow sensitivity for more global motions of the structure. Indeed, an early work on TRP phosphorescence [21] showed that the intrinsic decay rate was highly sensitive to the dynamic nature of the surrounding matrix, and suggested using this signal of embedded chromophores as a probe for the flexibility of the surrounding protein conformation at room temperature. The intrinsic decay rate has been determined by excluding the main quencher of TRP phosphorescence: molecular oxygen.

We reported a new phosphorescence-based method [22], in which we basically reversed this known approach. Instead of deoxygenation, we saturated the samples with oxygen, and registered the lifetime at various temperatures in thermal equilibrium from 10 K up to room temperature. In the frozen state of the protein matrix, the oxygen molecules cannot move, thus they cannot exert quenching on the phosphorescence of a marker molecule. The thermal activation of protein dynamics, however, gives rise to the diffusion of O_2_, leading to intensive phosphorescence quenching. This becomes a measurable signal for the dynamic properties of the matrix.

We considered it crucially important not to add any cryosolvents–such as glycerol–to the buffer containing the molecules. We consider this a fundamental difference between our technique and other low temperature optical spectroscopic methods applied for protein dynamics studies. Optical spectroscopy at low temperature generally uses a cryosolvent like glycerol as the standard method, adding it in quite high percentages (up to even more than 90%) [23–25] to the aqueous buffer of the protein solution to avoid light scattering, to extend the measurable range of kinetic parameters, and to avoid the possible structural effects of crystalline ice formation during cooling. Glycerol content up to 60% is acceptable, because most proteins maintain their substrate binding ability up to this concentration. We presented, however, in our previous study [22] that glycerol concentration above this level drastically modifies the experimental results. In our opinion, such experiments provide information more about glycerol than the protein. We showed that by not using cryosolvent, the ms dynamics of Hb was activated at around 200 K, while that of the aqueous solvent took place at an about 40–50 K higher temperature.

In this paper, we report results of phosphorescence lifetime experiments performed with the purpose of unraveling possible fine tuning effects of allosteric effectors on the conformational dynamics of human Hb. The measurements were based on the phosphorescence of Zn-protoporphyrin (Zn-PP) substituting the heme in the β subunits of hybrids of human Hb: [αFeO_2_]_2_-[β-Zn]_2_-HbA (abbreviated as Zn-HbA). The role of Zn-PP in this experiment is the same as TRP’s was, namely it is also quenched by oxygen diffusion. To monitor the global dynamics of the conformation, the temperature range of activating O_2_ diffusion was determined by measuring the phosphorescence lifetime in samples, saturated with oxygen, and equilibrated at various temperatures from cryogenic to room temperature. The average lifetime data as a function of temperature was analyzed. This way, the effect of dynamic motions in the ms time range was expected to be observed. We concentrated on the quenching activation at lower temperature (Transition1) at around 200 K, which is characteristic of Zn-HbA. In this work, we did not study the 2^nd^ transition step attributed to the aqueous solvent (see details in ref. [22]). We found that the thermodynamics of activation in Hb is sensitive to binding allosteric effectors.

The thermodynamic model used to describe this phase transition and the fitting to average lifetime data yielded the change of energy and entropy (Δ*E*, Δ*S*) in the activation process, and the quenching rate *K*_d_. *K*_d_ is the ratio by which the decay process is sped up due to dynamic quenching. The fitted parameters changed due to the presence of allosteric effectors in line with results of previous experimental studies. The correlation among the changes of oxygen affinity, the activation energy and entropy, and the subunit compressibility [1] suggests that allosteric effectors tightly couple the whole protein and point out the importance of global dynamics in the regulation of Hb function. This coupling to the global dynamics suggests that allostery generally per se is made possible by the existence of global structural motions (fluctuations), which encompass the whole (or a very large portion) of the protein molecule.

The "slow global motions" is a rather frequently used term in this paper, but we know it is not unambiguous in a way that as the matrix surrounding the protein is frozen, a quaternary transition cannot happen. However, if we consider the normal mode analysis as a suitable method to characterize intrinsic slow collective motions then there are low frequency modes, which span the entire quaternary structure.

## Materials and methods

### Chemicals

Chemicals such as sodium chloride (NaCl), 4-(2-hydroxyethyl)-1-piperazineethanesulfonic acid (HEPES), inositol hexaphosphate (IHP), 2,3-diphosphoglycerate (DPG) and bezafibrate (BZF), the highest purity available, were purchased from Sigma-Aldrich (St. Louis). Catalogue numbers are 68388 (IHP), D5764 (DPG), B7273 (BZF). All samples were prepared in 100 mM HEPES, pH 7.4 with double distilled water.

### Sample preparation

The experiments were performed on hybrid human HbA with Zn-PP substituting the heme in the β subunits, [αFeO_2_]_2_-[β-Zn]_2_-HbA (called as Zn-HbA) that were prepared in Prof. T. Yonetani’s laboratory at the University of Pennsylvania (Philadelphia, USA) as described in Ref. [26], and kindly provided for our experiments. These samples were stored at -80°C. After the thawing of Zn-HbA, allosteric effectors were added: IHP and DPG in a final concentration of 2 mM, BZF in 10 mM, and the NaCl concentration was 100 mM. These effector concentrations could ensure the saturation of the binding sites of 60 μM final Zn-HbA concentration in buffer solution (pH = 7.4). The sample quality was checked before experiments by recording an absorption spectrum in the 270–700 nm wavelength range. We emphasize that the samples did not contain cryosolvent (such as glycerol) at all.

### Experimental setup

A detailed description of the conditions in which the experiments were carried out can be found in our paper [22], a brief summary is provided below. The adjustment of the sample temperature from 10 K up to 273 K was done using a Cryophysics M22 type closed-cycle helium refrigerator (Cryophysics SA, Geneva, Switzerland) and a LakeShore M330 temperature controller (LakeShore, Inc., Westerville, USA). The sample of 80 μl volume was contained in a quartz (UV fused silica) tube of 2.7 mm inner diameter, sealed gastight with a conical Teflon stopper. The sample was saturated with the oxygen of air at room temperature by carefully and slowly bubbling air into the sample for 5 min, then it was quickly cooled down to 8 K using the normal “fast cooling” technique (without liquid nitrogen). The phosphorescence lifetime of the samples was determined along with a stepwise warming period starting from 10 K. The gradual heating took place in 10 or 5 K increments, and a 45-min period of thermal equilibration was sufficient at each temperature before the start of one decay measurement. The lifetime of Zn-PP was measured using the time-domain mode of an EAI CD900 spectrometer (Edinburgh Analytical Instruments, Edinburgh, U.K.) equipped with a μF900 Xe flashlamp of a power of approximately 1 mJ/pulse and a pulse width of 2 μs fwhm. Excitation and emission wavelengths were 409 and 723 nm, respectively, with 5 nm bandpass. The emission signal was very weak due to light scattering and quenching effects, up to 14000 consecutive flashes were summed with a time resolution of 80 μs to reach an acceptable signal/noise ratio for evaluation. Phosphorescence photons were detected by a photomultiplier tube (R928, Hamamatsu Photonics, Shimokanzo, Japan) operated in single photon counting mode and cooled to -18°C (C65972 cooler). After amplification, data were collected by a Norland 5000 multichannel analyzer card (Viking Instruments, Madison, U.S.A).

### Evaluation of phosphorescence decay

Decay process of Zn-PP molecules embedded into HbA cannot be characterized by a single lifetime due to both the heterogeneous conformational environment and the heterogeneity of the quenching conditions of the ensemble of protein molecules. Thus, we determined the average lifetime of the heterogeneous lifetime population, and used it as a dynamic parameter [22]. The decay data (*I*(*t*) functions) were fitted first with the sum of exponential decay functions of discrete lifetime values:

$$I(t)=\sum_{i=1}^{n}A_{i}e^{-t/\tau_{i}},$$ (1)

where *A*_*i*_ and *τ*_*i*_ are the amplitudes and lifetimes of the individual components, respectively, and *n* is the number of exponential functions used for the fitting. For further analysis the ensemble average <*τ* > was calculated as:

$$<\tau >\;=\frac{\sum_{i=1}^{n}A_{i}\tau_{i}^{2}}{\sum_{i=1}^{n}A_{i}\tau_{i}}.$$ (2)

## Results and discussion

### Normalized average phosphorescence lifetime of Zn-HbA as a function of temperature

**Fig 2:**
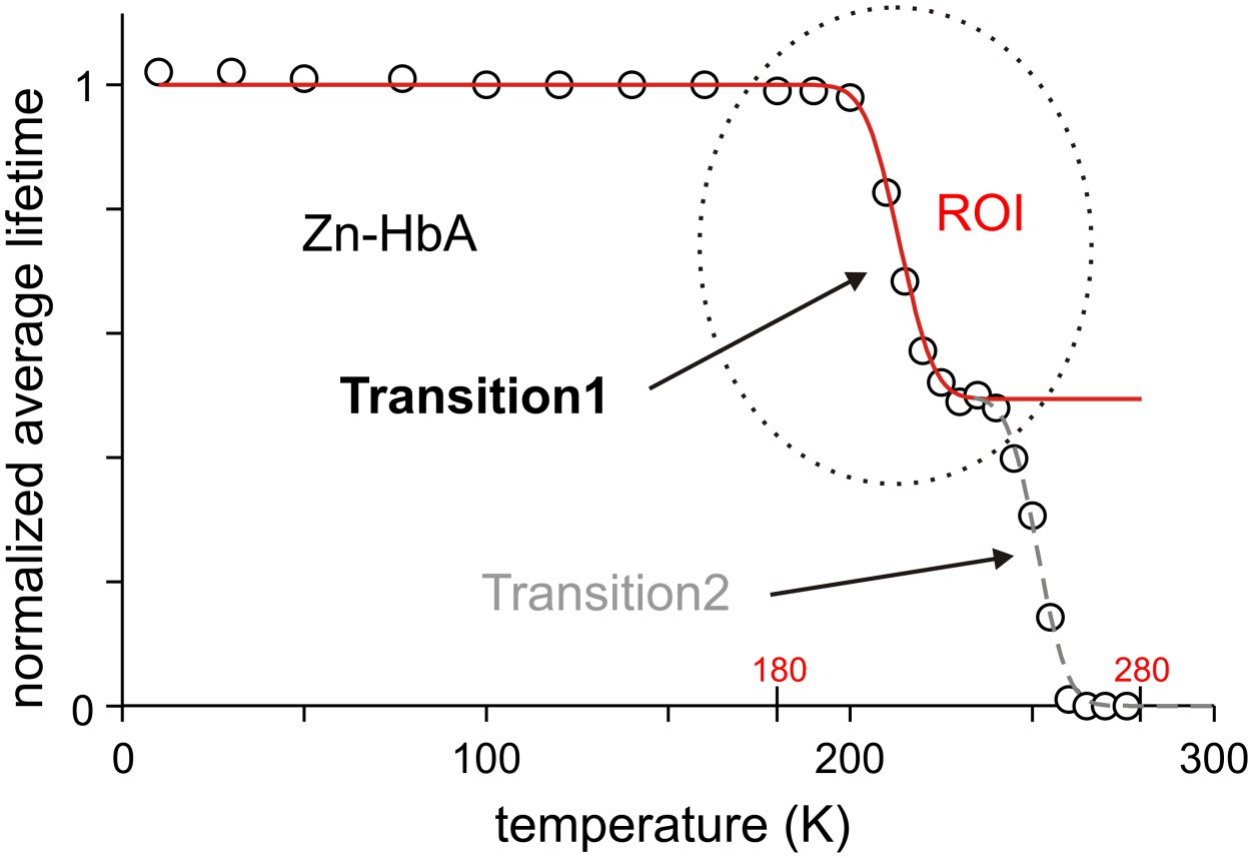
Normalized average phosphorescence lifetime values of Zn-HbA as a function of temperature measured in a stepwise warming process (open circles). Transition1 is marked as the region of interest (ROI). The continuous red curve corresponds to the fitted data points of Transition1 by our thermodynamic model. (Transition2 is marked with gray dashed curve).

We used Eq 1 for the fitting of decay curves. According to our experience, an acceptable least-squares fit required maximum 5 discrete exponential functions (*n* ≤ 5 see Eq 1). The ensemble average < τ > was calculated according to Eq 2. This value did not change significantly from 10 K up to about 180 K. Thus, the average over this temperature range of the slightly fluctuating lifetime was considered as a normalization factor, *τ*_0_, the characteristic reference lifetime of “frozen” state (without dynamic quenching). Normalized average phosphorescence lifetime values (< τ >/*τ*_0_) are plotted in Fig 2.

As Fig 2 shows, this ratio for Zn-HbA as a function of temperature has two markedly separated sharp drops above 180 K. They supposedly indicate that new additional quenching effects become activated at specific temperature ranges. As we experimentally verified, the first quenching step at around 200 K (Transition1) is characteristic for the protein, the second one is related to the aqueous solvent matrix [22]. As we have mentioned already, our samples did not contain a cryosolvent (like glycerol), which would have drastically affected the measurements and could have resulted in false results. In this work, we studied Transition1. In Fig 2, Transition1 is marked as the region of interest (ROI), to emphasize that the further evaluation of the measurements was done only in this range. Transition1 was fitted by a thermodynamic model outlined in the Appendix using the equation:

$$\frac{<\tau >}{\tau_{0}}(T)=\frac{1+e^{-(\Delta E-T\Delta S)/RT}}{1+K_{\text{d}}e^{-(\Delta E-T\Delta S)/RT}}.$$ (3)

Here, Δ*E* and Δ*S* are the molar energy and entropy differences between the “frozen” and “molten” or thermally activated states, *K*_d_ is the ratio by which the decay process is sped up due to dynamic quenching. The basic idea in the model was that conformational changes caused by the binding of allosteric effectors may influence the global dynamics of the HbA molecule, and this change may result in different activation energy (Δ*E*) and entropy (Δ*S*) values.

### Activation of global dynamics of Zn-HbA in presence of heterotropic allosteric effectors

We have chosen four different effectors (Cl^-^, IHP, DPG, BZF) added in saturating concentrations (based on the literature, and our previous work) [15, 27] to test the hypothesis mentioned above. The order of efficiency of effectors in lowering the oxygen affinity of HbA is known as: Cl^-^ < IHP ≈ DPG < BZF [11].

Table 1 shows the measured average phosphorescence lifetime (at low temperature without dynamic quenching) of “stripped” Zn-HbA–the term “stripped” meaning the lack of any allosteric effector bound–and that of those bound to allosteric effectors. It is observable that the deviation among the average values is less than the standard deviation of each lifetime. This means that the effector binding itself does not perturb directly the atomic environment of the Zn-heme (in the β subunits).

**Table 1:** The measured average phosphorescence lifetime (± SD) of stripped Zn-HbA and of those bound to allosteric effectors at low temperature without dynamic quenching.

Sample	*τ*_0_ (ms)
Zn-HbA stripped	28.7 ± 0.6
Zn-HbA + Cl^-^	28.9 ± 0.8
Zn-HbA + IHP	29.0 ± 0.8
Zn-HbA + DPG	27.6 ± 1.1
Zn-HbA + BZF	28.0 ± 0.9

**Fig 3:**
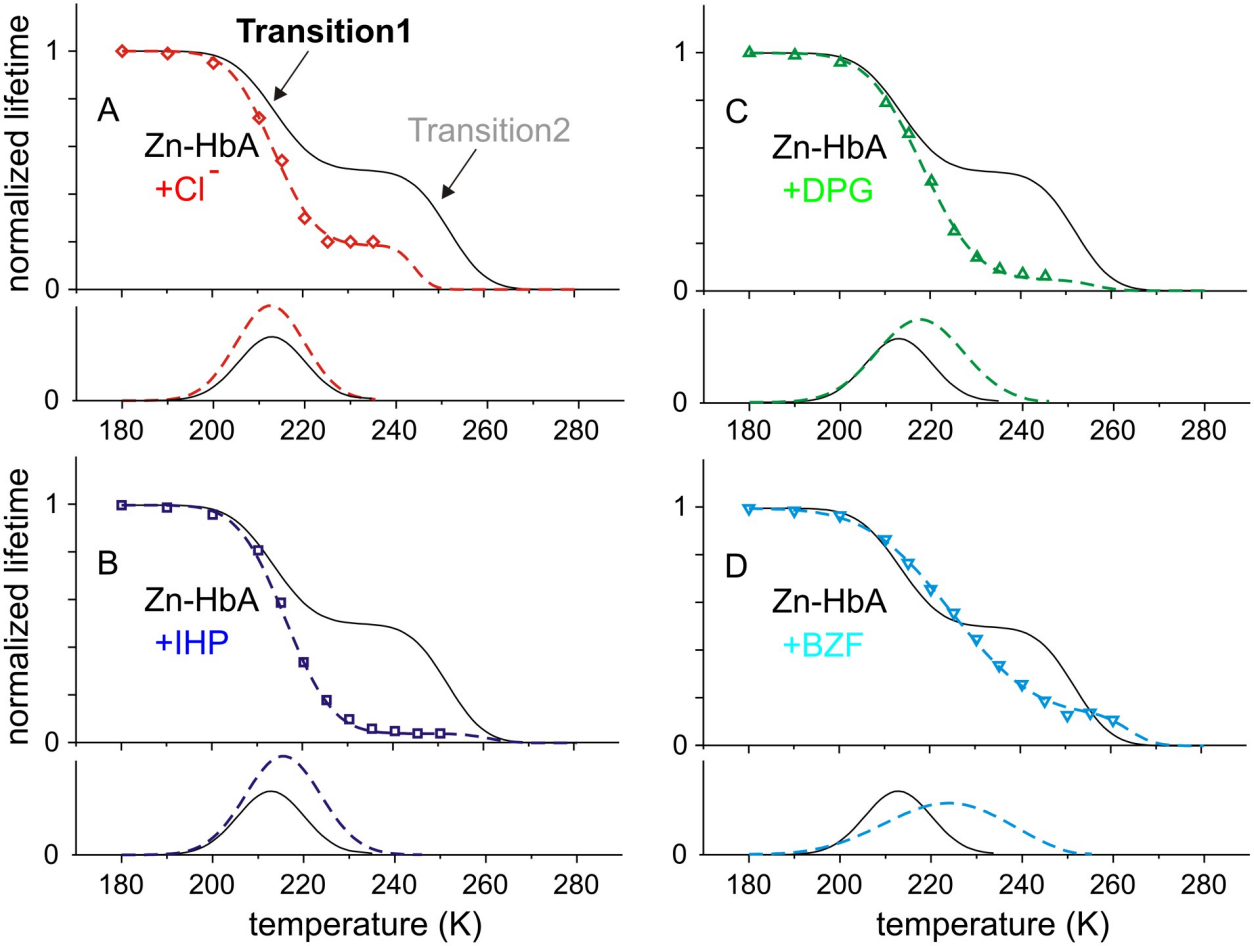
Normalized average phosphorescence lifetime values of Zn-HbA bound to different allosteric effectors as a function of temperature. Original data points are shown with symbols. Transition1 is marked with colors. The original, colored dashed curves are the fitting functions of the thermodynamic model: Zn-HbA bound to Cl^-^: red diamonds (A); to IHP: blue squares (B); to DPG: green upward triangles (C); to BZF: cyan downward triangles (D). For comparison, the Transition1 of stripped Zn-HbA is also shown in each graph with black curve, but Transition2 on it is marked with gray dashed curve. The negative derivative functions are also shown just below the presented dataset (bottom graphs).

Fig 3 shows the normalized average phosphorescence lifetime values of Zn-HbA bound to allosteric effectors as a function of temperature. It can be seen that the binding of allosteric effectors modifies the function, and significantly changes the first transition. While the binding of Cl^-^, IHP and DPG cause a relatively small change in the transition temperature, BZF causes a large shift accompanied by a similarly significant broadening of the transition range. Transition1 of original data points (colored symbols in Fig 3) was fitted by the thermodynamic model (colored dashed curves in Fig 3). The thermodynamic parameters calculated from the fitting of Eq 3 to the data are shown in Table 2. This transition was shown to be specific for the protein, therefore its change due to effector binding may be of biological significance.

**Table 2:** Thermodynamic parameters (± SD) of the fitting for all kinds of samples (based on our model, see S1 Appendix): Δ*E* (energy change), Δ*S* (entropy change), *K*_d_ (ratio by which the decay process is sped up due to dynamic quenching), *T*_MP_ (midpoint temperatures).

Sample	Δ*E* (kJ/mol)	Δ*S* (J/mol)	*K*_d_	*T*_MP_ (K)
Zn-HbA stripped	113.4 ± 4.4	526 ± 22	2.0 ± 0.1	213.1 ± 0.3
Zn-HbA + Cl^-^	97.7 ± 5.6	442 ± 29	5.3 ± 0.4	214.2 ± 0.7
Zn-HbA + IHP	77.4 ± 3.5	329 ± 24	31.5 ± 2.8	216.4 ± 0.4
Zn-HbA + DPG	71.5 ± 3.7	299 ± 27	29.4 ± 3.0	218.5 ± 0.6
Zn-HbA + BZF	42.3 ± 1.7	163 ± 10	17.8 ± 2.0	225.9 ± 2.5

### Analysis of the thermodynamic parameters

Both the activation energy and entropy values of the effector-bound cases become significantly lower than in the stripped case. Cl^-^ ions have a relatively moderate effect, while DPG and IHP decrease the values by about 30%, and BZF by more than 50%. It is worth mentioning that the order of the change in the Δ*E* and Δ*S* values corresponds to the effector strength: Cl^-^ < IHP ≈ DPG < BZF. That is, the parameters are related to the efficiency of O_2_ binding. We may convert the meaning of the results into the conclusion that O_2_ binding may require a certain “amplitude” of conformational dynamics, and the effectors exert their allosteric regulation by reducing this amplitude (see decrease in Δ*S*). In the case of dynamics with reduced amplitude, its energy of activation can also be supposedly lower (see reduced Δ*E*).

The change in the *K*_d_ value is an increase due to effector-binding. *K*_d_ itself is related to the mechanism of the quenching effect. We may think of collisional quenching between the electronic orbital of the triplet excited state of the Zn-heme and the diffusing O_2_. A change in the efficiency of this collision may result from a change in the diffusion coefficient of the quencher inside the protein, or from a change in the conformational condition of the collision resulting in the quenching effect. Since the effectors cause similar effects in *K*_d_, although they bind at different places that are distant from the heme, we cannot think of a general conformational effect on the heme-pocket. Consequently, we conclude that the effectors may act by increasing the diffusion rate through influencing the parameters of the overall dynamic fluctuations.

The parameters are interconnected by the formula:

$$T_{\text{MP}}=\frac{\Delta E}{\Delta S+R\;\text{ln}\;K_{\text{d}}}.$$ (4)

thus, the relative magnitude of Δ*E*, Δ*S* and *K*_*d*_ should change by the addition of effectors in a way that *T*_*MP*_ increases, as indicated by the measurements.

### A dynamic model for the quenching effect

The energy and entropy values derived from the data help us to construct a model for the diffusion of the quencher (O_2_). As we argue below, they are consistent with the opening and closing cavities of dynamic nature that form a passage for the quencher in the structure. The diffusion of oxygen molecules would take place by “hopping around” in the protein matrix, with a step size equal to the size of such a cavity. Taking the denaturation entropy [28] of approximately 35 cal/(mole K residue) ≈ 145 J/(mole K residue), the observed entropy change is equal to the denaturation entropy of 1… 4 residues. The energy change is in the order of a few H-bonds that may be broken along with cavity formation, thus the data are consistent with such an atomic model.

In the view of this, we may say that the activation of dynamic cavity formation is affected by binding the effectors making the quenching effect more efficient. The activation energy and entropy decreased, which is in line with increased efficiency, i.e. increased diffusion coefficient and increased *K*_*d*_.

### Global conformational dynamics as signal transmitter

As we discussed above our work experimentally demonstrates that dynamic changes are induced by the effectors. It is well accepted to interpret changes in conformational fluctuations based on the ensemble model. That is, the background of a change in conformational fluctuations arises from a change in the population of energy valleys of the rugged energy surface of the funnel model [29] of protein conformation. A recent crystallographic approach reveals that allosteric transition in hemoglobin involves population shifts in multiple quaternary conformers, and the structural data strongly suggest that a ligated hemoglobin exists in an ensemble of conformations [30]. These resulting ensembles undergo a population shift in response to the binding of an allosteric effector.

Several lines of evidence suggested discrete multi-state transition, which is expected to result from proteins being flexible entities, and crystal structures can only provide a subset of the conformational ensembles present under physiological conditions [31]. This is how we interpret the observed change in the quenching efficiency of Zn-HbA phosphorescence. The change in the dynamic parameters of the quenching process arises because the induced population shift in the conformational ensemble increases the contribution of new pathways of the quencher due to a realignment of the dynamic fluctuation patterns of the structure [32]. This realignment may then play a role in transmitting information through the interfaces between the monomeric subunits, where the effectors are bound, and may also serve as an information “pathway” through the entire structure. In this way, allosteric effectors may influence the function of Hb not only through static structural changes, but also through influence on the dynamic global movement patterns of the entire structure of the protein.

### The timescale of observed dynamics and the temperature of the experiment

With the optical method applied in this work [22], a special type of dynamics is monitored, namely, only the motions that have a characteristic time window of approximately 10^−5^… 1 seconds.

This slow dynamics of the protein correlate with its biological function in a sense that any biologically significant changes in the protein’s function also manifest themselves in changes of these activation parameters. But dynamics at low temperature are not identical to the dynamics of the same time scales at room temperature, as the boundary of the protein is bound to a solid matrix during Transition1. The exact differences between these limited dynamics and the dynamics under physiological conditions cannot be inferred to with this technique, unfortunately. However, some structural information can still be deduced if we consider the opening and closing of the channels as a means of a limited diffusion of oxygen molecules in the structure. Given the relation that the distance covered by a free diffusion is proportional to the square of time, the observed dynamic quenching comes from a square root distance weighed dynamics around the chromophore. These dynamics being affected by binding effector molecules at distant places (such as subunit interfaces) serve as proof for the significant role of global dynamics in the allosteric effect.

It is logical to compare the presented results with those obtained by earlier compressibility measurements on the same system [1]. Compressibility data were obtained by fluorescence line narrowing (FLN) spectroscopy on Zn-HbA, by monitoring the compressibility in the heme pocket. Compressibility is used to measure the variance of volume fluctuations. The range of sensitivity in case of ZnHbA is limited to the size of a subunit. In that case, the sample allowed for the characterization of both the α and the β subunits, and the effect of the same allosteric effectors was studied. We found an asymmetric effect, the change in the compressibility affected the β subunits more, and the relative change was significantly smaller than the change observed by the present phosphorescence method. Since FLN is a low temperature fluorescence technique, and is hence inherently sensitive to the dynamics of fluorescence timescale (ps-ns), we can conclude that the effectors cause a significantly larger change in slow dynamics than in ps-ns timescale dynamics. This suggests that while the binding primarily affects the residues in the narrow surrounding of the effector, this propagates through microsecond-or-longer timescale fluctuations throughout the entire structure. The view of relating our experimental technique to the characterization of global dynamics is strongly supported by comparing the thermal activation of phosphorescence quenching under two conditions [22]. In one case, the signal of ZnPP was measured, in the other case, the phosphorescence of the six TRP-s was registered. The close agreement of the two functional forms of Transition1 supports the interpretation that in both cases, the effect of global conformational fluctuations was recorded. Global fluctuations were also suggested based on in-silico molecular dynamics simulation [33, 34]. The results of the simulations, however, are not directly comparable with our data, since the technique needs to be extended to cover the ms timescale, to reveal the atomistic details of the coupled motions spanning the entire structure of the molecule [35].

One possible solution is the coarse graining method [36, 37]. These approaches are in line with the ensemble view of allostery. The motions described by such ensembles can be evaluated by normal mode analysis. The low-frequency modes (soft modes) are particularly relevant to allostery, as they are both highly cooperative and robustly defined by the overall architecture of the system. These soft modes are estimated computationally from experimental structures, using coarse-grained elastic network models, which strongly depend on the reference structure. Soft modes correspond to slow motions at a longer timescale.

Similarly, coarse graining is usable for higher-order cooperativities (HOCs) that may arise through allostery, in which binding is collectively modulated by multiple other binding events [38]. Using this method, sufficiently complex ensembles can implement any form of information integration achievable without energy expenditure, including all patterns of HOCs. Several approaches depend on the size and complexity of the systems under study, making it difficult to evaluate the information they provide about the identified transition paths [39].

All these findings confirm the relevance of our method, where we measure the ensemble averages, and conclude that allosteric effectors tightly couple the whole protein, and point out the importance of global slow dynamics in the regulation of Hb function. In addition, our results strongly suggest that a simple mechanistic model may be inadequate to explain the allosteric effects. Instead, specific motional patterns–most probably spanning the entire tertiary structure through inter-domain couplings–may be responsible for the change in oxygen affinity.

## Conclusions

Global, slow dynamics may play an important role in the regulation of protein function. It may even play a major role in allostery. If we think about the global dynamics as some sort of collective oscillations of the structure, then a larger assembly like HbA may have many more types of such oscillations than a relatively small structure like Mb. Also, if a protein is small, a significant part of its structure is only a few Å-s away from the solvent, and solvent effects may influence its dynamics more strongly. Thus, it may not be “autonomous enough” to build up a sufficient, complex internal dynamics, which would allow for allosteric fine-tuning. This hypothesis as a generalized conclusion could also be tested in the future by the same elaborated phosphorescence method applied on other proteins.

## Supporting information

S1 Appendix (DOCX)

## References

[1] Schay G, Kaposi AD, Smeller L, Szigeti K, Fidy J, Herenyi L. Dissimilar flexibility of α and β subunits of human adult hemoglobin influences the protein dynamics and its alteration induced by allosteric effectors. PLoS ONE. 2018;13(3): e0194994. doi: 10.1371/journal.pone.0194994 29584765 PMC5871000

[2] Monod J, Wyman J, Changeux JP. On the nature of allosteric transitions: A plausible model. J Mol Biol. 1965;12: 88–118. doi: 10.1016/s0022-2836(65)80285-6 14343300

[3] Viappiani C, Abbruzzetti S, Ronda L, Bettati S, Henry ER, Mozzarelli A, et al. Experimental basis for a new allosteric model for multisubunit proteins. Proc Natl Acad Sci USA. 2014;111: 12758–12763. doi: 10.1073/pnas.1413566111 25139985 PMC4156698

[4] Ackers GK, Holt JM. Asymmetric cooperativity in a symmetric tetramer: Human hemoglobin. J Biol Chem. 2006;281: 11441–11443. doi: 10.1074/jbc.R500019200 16423822

[5] Perutz MF. Stereochemistry of cooperative effects in haemoglobin. Nature. 1970;228: 726–739. doi: 10.1038/228726a0 5528785

[6] Koshland DE, Nemethy G, Filmer D. Comparison of experimental binding data and theoretical models in proteins containing subunits. Biochemistry. 1966;5: 365–385. doi: 10.1021/bi00865a047 5938952

[7] Motlagh HN, Wrabl JO, Li L, Hilser VJ. The ensemble nature of allostery. Nature. 2014;508: 331–339. doi: 10.1038/nature13001 24740064 PMC4224315

[8] Ribeiro AAST, Ortiz V. A chemical perspective on allostery. Chem Rev. 2016;116: 6488–6502. doi: 10.1021/acs.chemrev.5b00543 26741913

[9] Henry ER, Bettati S, Hofrichter J, Eaton WA. A tertiary two-state allosteric model for hemoglobin. Biophys Chem. 2002;98: 149–164. doi: 10.1016/s0301-4622(02)00091-1 12128196

[10] Tsuneshige A, Park S, Yonetani T. Heterotropic effectors control the hemoglobin function by interacting with its T and R states—a new view on the principle of allostery. Biophys Chem. 2002;98: 49–63. doi: 10.1016/s0301-4622(02)00084-4 12128189

[11] Yonetani T, Park S, Tsuneshige A, Imai K, Kanaori K. Global allostery model of hemoglobin. J Biol Chem. 2002;277: 34508–34520. 12107163 10.1074/jbc.M203135200

[12] Nussinov R, Tsai CJ. Allostery without a conformational change? Revisiting the paradigm. Curr Opin Struct Biol. 2015;30: 17–24. doi: 10.1016/j.sbi.2014.11.005 25500675

[13] Tsai CJ, Nussinov R. A unified view of “How allostery works”. PLoS Comput Biol. 2014; 10(2): e1003394. doi: 10.1371/journal.pcbi.1003394 24516370 PMC3916236

[14] Laberge M, Kövesi I, Yonetani T, Fidy J. R-state hemoglobin bound to heterotropic effectors: models of the DPG, IHP and RSR13 binding sites. FEBS Lett. 2005;579: 627–632. doi: 10.1016/j.febslet.2004.12.033 15670819

[15] Kövesi I, Schay G, Yonetani T, Laberge M, Fidy J. High pressure reveals that the stability of interdimeric contacts in the R- and T-state of HbA is influenced by allosteric effectors: Insights from computational simulations. Biochim Biophys Acta. 2006;1764: 516–521. doi: 10.1016/j.bbapap.2005.12.002 16427817

[16] Arnone A. X-ray diffraction study of binding of 2,3-diphosphoglycerate to human deoxyhaemoglobin. Nature. 1972;237(5351): 146–149. doi: 10.1038/237146a0 4555506

[17] Imai K. Allosteric Effects in Hemoglobin. Cambridge University Press, 1982.

[18] Poyart C, Marden MC, Kister J. Bezafibrate derivatives as potent effectors of hemoglobin. Methods Enzymol. 1994;232: 496–513. doi: 10.1016/0076-6879(94)32062-4 8057877

[19] Lakowicz JR, Weber G. Quenching of fluorescence by oxygen. A probe for structural fluctuations in macromolecules. Biochemistry. 1973;12: 4161–4170. doi: 10.1021/bi00745a020 4795686 PMC6959846

[20] Lakowicz JR, Weber G. Quenching of protein fluorescence by oxygen. Detection of structural fluctuations in proteins in the nanosecond time scale. Biochemistry. 1973;12: 4171–4179. 4200894 10.1021/bi00745a021 PMC6945976

[21] Strambini GB, Gonnelli M. The indole nuclens triplet-state lifetime and its dependence on solvent microviscosity. Chem. Phys. Lett. 1985;115: 196–200.

[22] Schay G, Herényi L, Kellermayer M, Módos K, Yonetani T, Fidy J. Millisecond time-scale protein dynamics exists prior to the activation of the bulk solvent matrix. J Phys Chem B. 2011;115: 5707–5715. doi: 10.1021/jp106755t 21395276

[23] Fenimore PW, Frauenfelder H, McMahon BH, Parak FG. Slaving: Solvent fluctuations dominate protein dynamics and functions. Proc Natl Acad Sci USA. 2002;99: 16047–16051. doi: 10.1073/pnas.212637899 12444262 PMC138562

[24] Fenimore PW, Frauenfelder H, McMahon BH, Young RD. Bulk-solvent and hydration-shell fluctuations, similar to α- and β-fluctuations in glasses, control protein motions and functions. Proc Natl Acad Sci USA. 2004;101: 14408–14413. 15448207 10.1073/pnas.0405573101 PMC521939

[25] Frauenfelder H, Fenimore PW, Chen G, McMahon BH. Protein folding is slaved to solvent motions. Proc Natl Acad Sci USA. 2006;103: 15469–15472. doi: 10.1073/pnas.0607168103 17030792 PMC1592535

[26] Tsuneshige A, Yonetani T. Preparation of mixed metal hybrids. Methods in Enzymology. 1994;231: 215–222. doi: 10.1016/0076-6879(94)31015-7 8041253

[27] Schay G, Smeller L, Tsuneshige A, Yonetani T, Fidy J. Allosteric effectors influence the tetramer stability of both R-and T-states of hemoglobin a. J Biol Chem. 2006;281(36): 25972–25983. doi: 10.1074/jbc.M604216200 16822864

[28] Karplus M, Ichiye T, Pettitt B. Configurational entropy of native proteins. Biophys J. 1987;52(6): 1083–1085. doi: 10.1016/S0006-3495(87)83303-9 3427197 PMC1330109

[29] Dill K, Chan H. From Levinthal to pathways to funnels. Nat Struct Mol Biol. 1997;4: 10–19. doi: 10.1038/nsb0197-10 8989315

[30] Shibayama N. Allosteric transitions in hemoglobin revisited. Biochim Biophys Acta Gen Subj. 2020;1864: 129335. doi: 10.1016/j.bbagen.2019.03.021 30951803

[31] Ahmed MH, Ghatge MS, Safo MK. Hemoglobin: Structure, Function and Allostery. Subcell Biochem. 2020;94: 345–382. doi: 10.1007/978-3-030-41769-7_14 32189307 PMC7370311

[32] Mouawad L, Maréchal JD, Perahia D. Internal cavities and ligand passageways in human hemoglobin characterized by molecular dynamics simulations. Biochim Biophys Acta. 2005;1724: 385–393. doi: 10.1016/j.bbagen.2005.05.014 15963643

[33] Hub JS, Kubitzki MB, De Groot BL. Spontaneous quaternary and tertiary TR transitions of human hemoglobin in molecular dynamics simulation. PLoS Comput Biol. 2010;6(5): p.e1000774. doi: 10.1371/journal.pcbi.1000774 20463873 PMC2865513

[34] Mouawad L, Perahia D, Robert CH, Guilbert C. New insights into the allosteric mechanism of human hemoglobin from molecular dynamics simulations. Biophys J. 2002;82(6): 3224–3245. doi: 10.1016/S0006-3495(02)75665-8 12023247 PMC1302112

[35] Costa MGS, Batista PR, Bisch PM, Perahia D. Exploring Free Energy Landscapes of Large Conformational Changes: Molecular Dynamics with Excited Normal Modes. J Chem Theory Comput. 2015;11: 2755–2767. doi: 10.1021/acs.jctc.5b00003 26575568

[36] Bahar I, Rader AJ. Coarse-grained normal mode analysis in structural biology. Curr Opin Struct Biol 2005;15: 586–592. doi: 10.1016/j.sbi.2005.08.007 16143512 PMC1482533

[37] Tama F, Brooks CL. Symmetry, form, and shape: guiding principles for robustness in macromolecular machines. Annu Rev Biophys Biomol Struct. 2006;35: 115–133. doi: 10.1146/annurev.biophys.35.040405.102010 16689630

[38] Biddle JW, Martinez-Corral R, Wong F, Gunawardena J. Allosteric conformational ensembles have unlimited capacity for integrating information. eLife. 2021;10:e65498. doi: 10.7554/eLife.65498 34106049 PMC8189718

[39] Wodak SJ, Paci E, Dokholyan NV, Berezovsky IN, Horovitz A, Li J, et al. Allostery in Its Many Disguises: From Theory to Applications. Structure. 2019;27: 566–578. doi: 10.1016/j.str.2019.01.003 30744993 PMC6688844

