# Supplementary material for: Slow dynamics measured by phosphorescence lifetime reveals global conformational changes in human adult hemoglobin induced by allosteric effectors
Source: PLoS One. 2022 Dec 1;17(12):e0278417. doi: 10.1371/journal.pone.0278417 (PMC9714750; doi:10.1371/journal.pone.0278417)
Supplement: S1 Appendix — (DOCX) [file pone.0278417.s001.docx]

**Appendix**

Newer thermodynamic model for Transition1.

In our earlier work, we suggested a simple thermodynamic model for describing the quenching effect due to protein dynamics activation [22]. In the newer, more plausible model, we distinguish a “frozen” state with a characteristic lifetime of *τ*_0_ (without dynamic quenching) and a “molten” state with a shorter characteristic lifetime of *τ*_d_ due to dynamic quenching effect, mainly because of the diffusing O_2_. The “molten” state corresponds to activated collective dynamics in the protein. In this state, the original lifetime – given as *τ*_0_= 1/*k*_0_ (where *k*_0_ is the respective rate constant) – will decrease due to appearing a new rate constant *k*_+_ in the denominator:

. (App1)

From this equation:

$k_{+}\tau_{0}=\frac{\tau_{0}}{\tau_{\text{d}}}-1=K_{\text{d}}-1=K_{\text{SV}}$, (App2)

where *K*_SV_ can be considered as a Stern-Volmer constant, which is proportional to the diffusion coefficient (*D*) of the quencher (O_2_), and which equals 0 in the “frozen” state. *K*_d_ = *τ*_0_/*τ*_d_ is the ratio by which the decay process is sped up due to dynamic quenching. Following this concept, the rate constant calculated from the average lifetime is given by:

, (App3)

where *n*_d_ from our original model is given in the usual form:

$\frac{n_{\text{d}}}{1-n_{\text{d}}}=e^{-(\Delta E-T\Delta S)/RT}$, (App4)

thus $n_{\text{d}}=\frac{e^{-(\Delta E-T\Delta S)/RT}}{1+e^{-(\Delta E-T\Delta S)/RT}}$. (App5)

Here, Δ*E* and Δ*S* are the molar energy and entropy differences between the “frozen” and “molten” or thermally activated states.

From equation App3 and App2:

, (App6)

and thus

$\frac{<\tau>}{\tau_{0}}=\frac{1}{1+n_{\text{d}}(K_{\text{d}}-1)}=\frac{1+e^{-(\Delta E-T\Delta S)/RT}}{1+K_{\text{d}}e^{-(\Delta E-T\Delta S)/RT}}$. (App7)

This is the new modified equation which was used for fitting procedure in this work.
